# CHARGE Syndrome in a Six-Month-Old Male Infant: A Case Report

**DOI:** 10.7759/cureus.84181

**Published:** 2025-05-15

**Authors:** Kawtar El Ouassifi, Anas Douami, Hind Ouair, Ibtihal Benhsaien, Aziz Bousfiha, Jalila El Bakkouri, Siham Salam, Fatima Ailal

**Affiliations:** 1 Allergy and Immunology, Pediatric Infectious Diseases and Clinical Immunology Unit, Abderrahim Harouchi Mother-Child Hospital, Ibn Rochd University Hospital Center, Casablanca, MAR; 2 Pediatric Radiology, Abderrahim Harouchi Mother-Child Hospital, Ibn Rochd University Hospital Center, Casablanca, MAR; 3 Medicine and Pharmacy, Laboratory of Clinical Immunology, Allergy, and Inflammation (LICIA) Hassan II University, Casablanca, MAR; 4 Biology, Immunology Laboratory, Ibn Rochd University Hospital Center, Casablanca, MAR

**Keywords:** charge syndrome, congenital coloboma, developmental delay, pediatric genetics, ventricular septal defect

## Abstract

CHARGE syndrome, short for coloboma, heart defects, atresia choanae, growth retardation, genital abnormalities, and ear anomalies, is a rare congenital disorder caused by genetic mutations. The diagnosis is based on a combination of major criteria, such as coloboma of the iris or choroid, choanal atresia, and hypoplastic semicircular canal, and minor criteria, including rhombencephalic dysfunction, hypothalamic-pituitary dysfunction, and malformations of the middle or external ear. Additional associated anomalies include malformations of mediastinal organs (heart, esophagus) and intellectual disability. We present the case of a six-month-old infant who showed signs of facial dysmorphism, delayed growth, and impaired psychomotor development. Ophthalmologic evaluation revealed chorioretinal coloboma. Cardiac ultrasound revealed a persistent ventricular septal defect. Based on the combination of the clinical findings, CHARGE syndrome was suspected and subsequently confirmed by genetic testing.

## Introduction

CHARGE syndrome, short for coloboma, heart defects, atresia choanae, growth retardation, genital abnormalities, and ear anomalies, is a rare genetic disorder with autosomal dominant inheritance [1]. It is characterized by a wide spectrum of clinical manifestations, including congenital heart defects, ocular anomalies, choanal atresia, genitourinary malformations, and psychomotor growth retardation [2]. In approximately two-thirds of cases, it is associated with a de novo mutation in the CHD7 gene [3].

In this report, we present the case of an infant diagnosed with CHARGE syndrome who also exhibited a primary immunodeficiency. The case was managed at the Pediatric Infectious Diseases and Clinical Immunology Unit of the Abderrahim Harouchi Mother-Child Hospital in Casablanca, Morocco.

## Case presentation

A six-month-old male infant, born to consanguineous parents, was hospitalized for persistent wheezing over two weeks. Clinical evaluation revealed developmental delay along with distinct craniofacial features, including retrognathia, a broad nasal bridge, a prominent forehead, and low-set ears. Auscultation identified bilateral wheezing and a systolic cardiac murmur. Additional findings included patent choanae and bilateral undescended testes.

**Figure 1:**
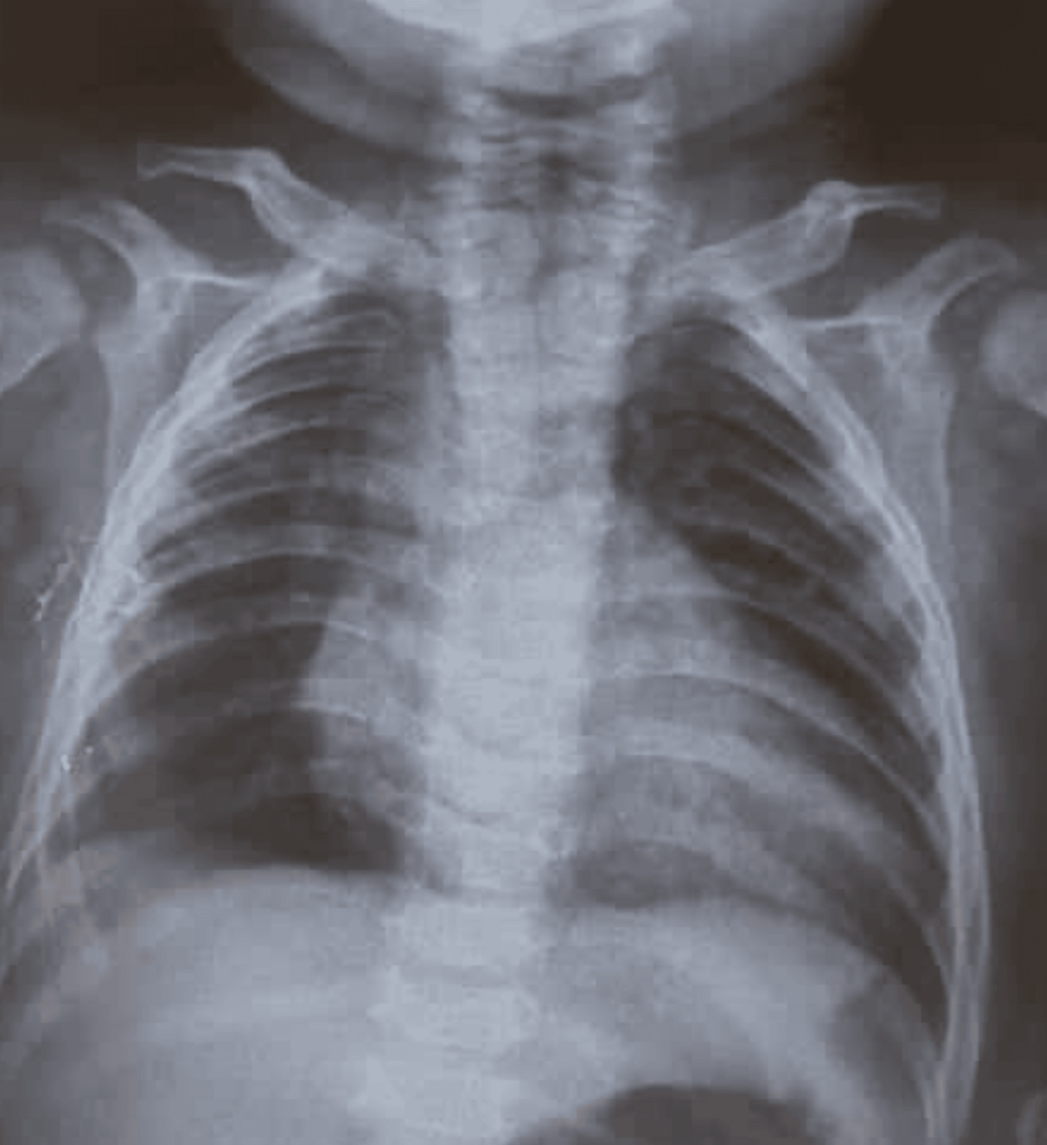
Chest radiography showing a boot-shaped cardiac silhouette

**Figure 2:**
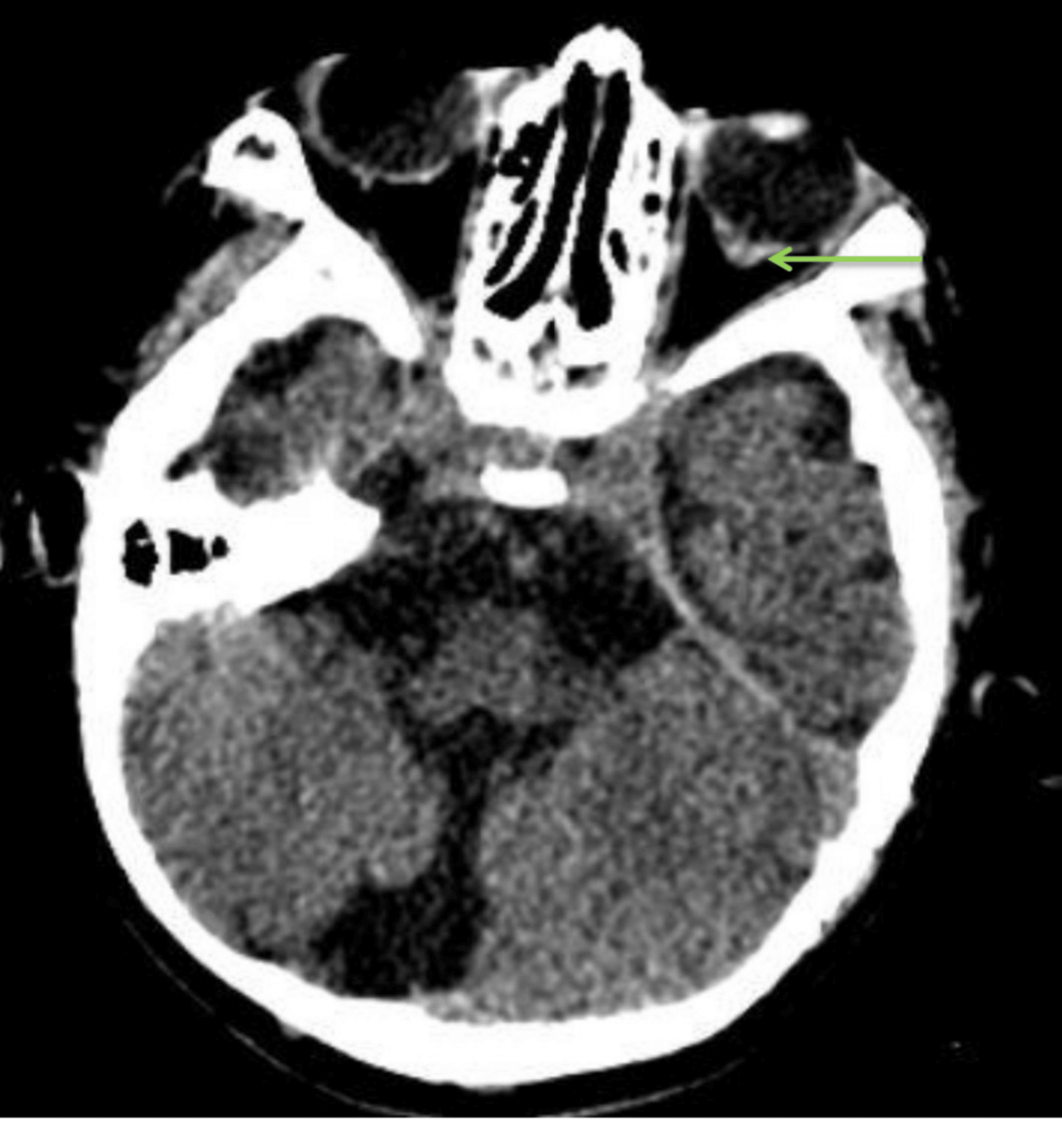
Posterior parietal defect of the eyeball (green arrow)

**Figure 3:**
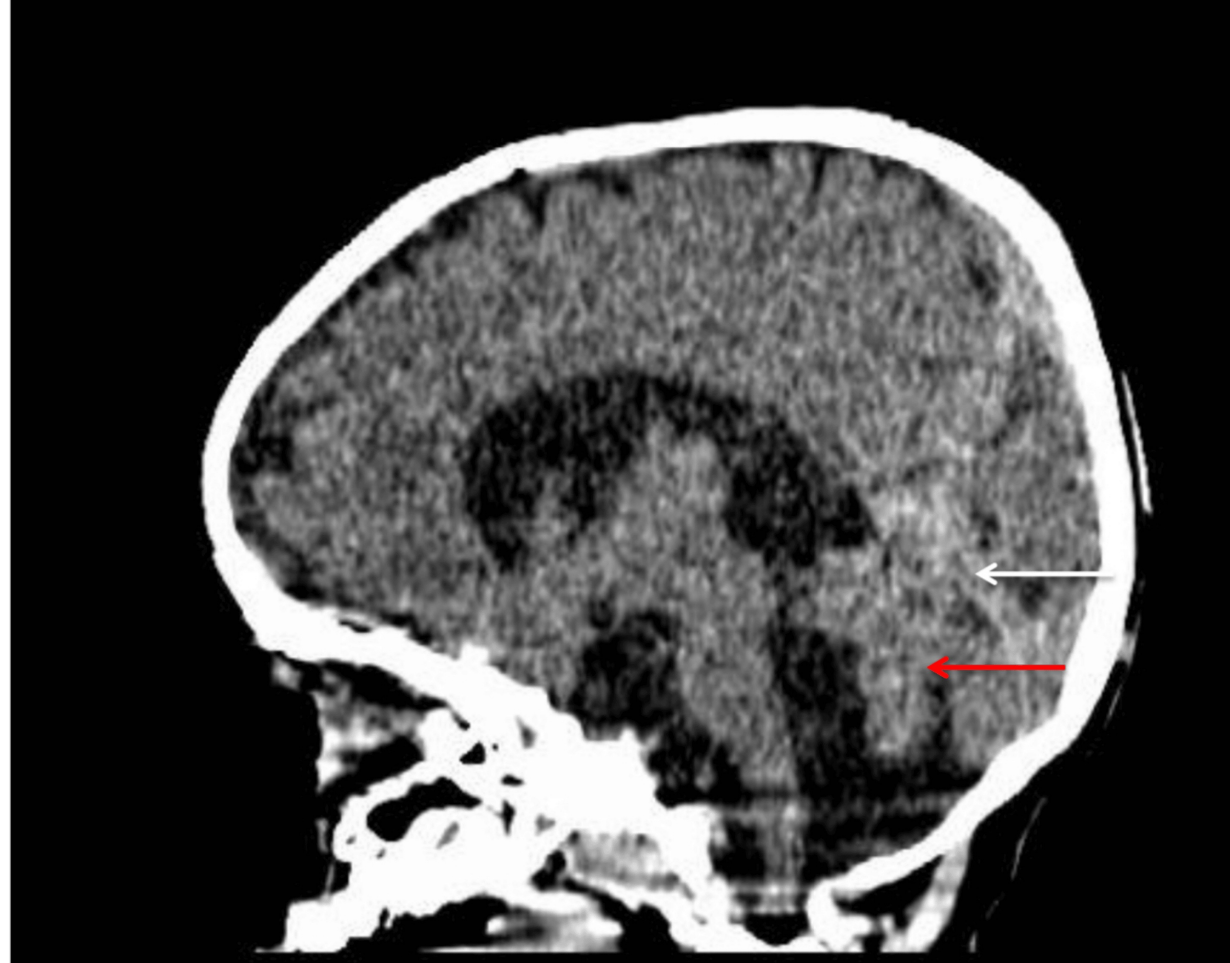
Sagittal section of the CT scan showing vermian hypoplasia (red arrow) associated with ascension of the cerebellar tent (white arrow)

Chest radiography showed right lower lobe pneumonia with diffuse interstitial infiltrates and a boot-shaped cardiac silhouette; the cardiothoracic ratio was elevated at 0.7 (Figure 1). Echocardiographic examination demonstrated a 5 mm patent ductus arteriosus with a left-to-right shunt and evidence of left ventricular enlargement. Scrotal ultrasound confirmed the diagnosis of bilateral cryptorchidism. Ophthalmological evaluation, including ocular ultrasonography, identified a chorioretinal coloboma in the left eye. Brain computed tomography revealed a 4 mm vitreous hernia (Figure 2) associated with a Dandy-Walker variant (Figure 3). Otolaryngologic examination and auditory findings were normal.

Laboratory investigations revealed lymphopenia (1499 cells/mm³) on complete blood count. Despite a normal-sized thymus on ultrasound, HIV serology was negative, immunologic profiling showed reduced lymphocyte subpopulations, with CD4 count at 1094/mm³, CD8 at 90/mm³, and CD19 at 138/mm³, and HLA-DR expression was normal (Table 1). A CHD7 c.776del (p.Glu2593Asnfs*3) mutation was confirmed via genetic testing.

**Table 1:** Lymphocyte subpopulation counts

Parameter	Patient’s values	Normal range
Total lymphocytes	1,499 /mm³	3,000–9,500 /mm³
CD4+ T cells	1,094 /mm³	1,500–3,500 /mm³
CD8+ T cells	90 /mm³	500–2,000 /mm³
CD19+ B cells	138 /mm³	300–1,200 /mm³
HLA-DR expression	Normal	Normal

The patient was managed with monthly intravenous immunoglobulin replacement therapy, prophylactic cotrimoxazole, surgical closure of the ductus arteriosus, and regular psychomotor rehabilitation. At two years of age, follow-up indicated ongoing failure to thrive, with persistent growth delay (weight: 10 kg) and continued psychomotor developmental delay.

## Discussion

The term CHARGE was first coined in 1981 to denote the key clinical features associated with the syndrome: coloboma, heart defects, atresia of the choanae, growth and developmental retardation, genital anomalies, and ear abnormalities [1]. The estimated incidence is around one in 10,000 live births, with no preference for sex [2]. Most instances are sporadic, with approximately 97% of cases attributed to de novo mutations in the CHD7 gene [4]. Diagnosis should be evaluated in any newborn exhibiting coloboma, choanal atresia, facial palsy, or distinct ear malformations, especially when accompanied by other congenital anomalies [5]. The approach to management is inherently multidisciplinary, engaging specialists from immunology, cardiology, otolaryngology, and endocrinology [4].

As noted by Blake et al. [1], clinical diagnosis necessitates the presence of either four major criteria or three major criteria along with three minor criteria.

Verloes states that a definitive diagnosis of typical CHARGE syndrome requires the fulfillment of all three major criteria or two major and two minor criteria [6]. For partial and atypical presentations, fewer combinations suffice for diagnosis. Genetic confirmation through the identification of a CHD7 mutation further substantiates the diagnosis.

Congenital heart defects linked to CHARGE syndrome exhibit a wide range of severity, from minor irregularities to significant defects that necessitate surgical intervention [7]. An analysis of 943 cases revealed that 76.6% of the patients had congenital heart defects: 26% presented with patent ductus arteriosus, 21% had ventricular anomalies, 18% experienced atrial septal defects, 11% were diagnosed with tetralogy of Fallot, and 24% had aortic arch anomalies [8].

The CHD7 gene produces a chromodomain helicase DNA-binding protein that plays a role in ATP-dependent chromatin remodeling. This protein interacts with methylated H3K4 and is pivotal in the development and movement of neural crest cells as well as cardiac mesodermal lineages [3].

Ocular manifestations are often characterized by coloboma, which is the most prevalent ocular defect associated with CHARGE syndrome. In our second patient, a chorioretinal coloboma was observed in the left eye, aligning with existing literature [9, 10]. Retinal colobomas are encountered more frequently than iris colobomas and can occur even if the eye appears normal from an external perspective. Notably, severe chorioretinal colobomas can be linked to microphthalmia [1].

Choanal atresia and other upper airway malformations are also described. Bilateral choanal atresia in neonates represents a medical emergency, potentially life-threatening if unrecognized [5]. Obstruction of the upper airway below the choanae is seen in 70% of patients, with 40% presenting with laryngomalacia, 20% with tracheomalacia, and 10% with subglottic stenosis. Additionally, 15% to 20% may present with cleft lip/palate or tracheoesophageal fistula [11]. Prenatal polyhydramnios may raise suspicion, especially when associated with conotruncal heart anomalies [5].

Immunodeficiencies associated with CHARGE syndrome are mainly a result of thymic underdevelopment, which results in inadequate T-cell production [12]. Lymphopenia is often more prevalent during infancy but may improve as the child grows. However, ongoing lymphopenia could be indicative of DiGeorge-like syndromes [13]. The most prevalent types of infections seen in these patients are respiratory and related to the ears, nose, and throat (ENT) [14]. T-cell abnormalities can affect subsets such as CD3⁺, CD4⁺, or CD8⁺. Although isolated antibody deficiencies are uncommon, IgA deficiency is the most frequently noted. Severe combined immunodeficiency is infrequent but has been reported [12].

Conducting an immunological assessment in individuals with CHARGE syndrome is crucial, not only for evaluating their immune function but also for customizing treatment strategies and monitoring potential risks [13].

The clinical course of CHARGE syndrome often includes severe medical complications such as respiratory infections, cardiac anomalies, and gastrointestinal malformations. Long-term management requires attention to comorbidities, prevention of complications, and enhancement of life quality [3, 13].

## Conclusions

CHARGE syndrome is a rare and complex genetic disorder that necessitates multidisciplinary management, including pediatrics, immunology, ENT specialists, and physiotherapists. Clinical variability, frequent comorbidities, and specific educational and rehabilitative needs make management particularly challenging. Nonetheless, recent advances in molecular genetics and personalized medicine offer promising prospects for improving both the prognosis and the quality of care in CHARGE syndrome. Ongoing multidisciplinary follow-up is crucial to meet patients’ evolving needs and to enhance their quality of life.
